# Creatine as a mitochondrial theranostic in predictive, preventive, and personalized medicine

**DOI:** 10.1007/s13167-025-00420-9

**Published:** 2025-09-01

**Authors:** Sergej M. Ostojic, László Rátgéber

**Affiliations:** 1 Faculty of Health Sciences, University of Pecs, Pecs, Hungary; 2 Department of Nutrition and Public Health, University of Agder, Kristiansand, Norway

**Keywords:** Theranostics, Creatine, Mitochondria, Bioenergetics, Metabolic, Neurodegenerative, Predictive preventive personalized medicine (PPPM / 3PM), Wearable biosensors, Digital health platform, Real-time monitoring, Sports nutrition

## Abstract

Creatine, traditionally recognized for its role in skeletal muscle energy metabolism, is increasingly emerging as a mitochondria-targeted theranostic agent with significant relevance to the framework of predictive, preventive, and personalized medicine (PPPM). However, several critical gaps currently limit its translation into clinical practice: (1) the lack of sensitive and standardized biomarkers for early detection of bioenergetic deficits, (2) limited incorporation of creatine profiling into predictive risk models, (3) insufficient personalization of supplementation strategies despite known interindividual variability in transporter function, endogenous synthesis, and tissue kinetics, and (4) underdeveloped clinical validation of advanced creatine formulations and delivery systems. This mini review addresses these unmet needs by consolidating evidence on creatine’s multifaceted biological functions—including stabilization of mitochondrial membranes, regulation of oxidative stress, support of mitochondrial biogenesis, and modulation of apoptotic signaling—across physiological and pathological states. By sustaining ATP homeostasis via the creatine kinase–phosphocreatine system and influencing mitochondrial dynamics and redox balance, creatine represents both a therapeutic and diagnostic candidate for diseases characterized by impaired bioenergetics. From a PPPM perspective, creatine profiling through biofluids, tissue sampling, and advanced imaging (e.g., proton magnetic resonance spectroscopy) offers a minimally invasive approach for early detection, patient stratification, and monitoring of mitochondrial function. Personalized intervention strategies—guided by molecular and phenotypic profiling—have the potential to maximize efficacy and minimize risk, while creatine loading or depletion tests may serve as functional biomarkers of mitochondrial reserve capacity and supplementation responsiveness. Finally, integration of creatine-centered diagnostics and therapeutics with multi-omics data, computational modeling, and digital health monitoring could overcome existing translational barriers. By reframing creatine from a sports nutrition supplement to a scalable, safe, and cost-effective component of mitochondrial medicine, this review outlines a pathway to address current diagnostic, predictive, and therapeutic deficits, ultimately supporting proactive, systems-level approaches to health maintenance and disease prevention.

## Introduction

The framework of predictive, preventive, and personalized medicine (PPPM) represents a paradigm shift from reactive disease management toward proactive healthcare, emphasizing early risk assessment, individualized interventions, and long-term health preservation [1]. A central challenge in operationalizing PPPM lies in identifying subtle, early-stage pathophysiological changes and integrating molecularly precise biomarkers into predictive models that can reliably stratify individuals by their future health trajectories. Mitochondria have emerged as pivotal targets within this paradigm because of their fundamental roles in bioenergetics, redox signaling, inflammation, and apoptosis [2–4]. As a result, there is growing demand for bioactive compounds that can both modulate mitochondrial function and serve as reliable, quantifiable indicators of mitochondrial health. Creatine (methylguanidinoacetic acid), traditionally recognized for its role in adenosine triphosphate (ATP) regeneration via the phosphocreatine energy shuttle, is now being repositioned as a mitochondria-targeted theranostic agent with significant potential for PPPM integration [5–7]. In addition to its classical role in skeletal muscle and brain energetics, creatine demonstrates multiple cytoprotective and bioenergetic effects at the mitochondrial level—stabilizing membrane potential, attenuating reactive oxygen species (ROS), preventing mitochondrial permeability transition pore (mPTP) opening, and regulating mitochondrial biogenesis, dynamics, and calcium buffering [8]. These pleiotropic effects make creatine an attractive candidate for both early detection and targeted treatment of disorders underpinned by mitochondrial dysfunction, including neurodegenerative diseases, metabolic syndromes, cardiovascular disorders, and age-related pathologies [9]. Yet, several critical deficits currently hinder the systematic incorporation of creatine into PPPM practice:

1. Diagnostic deficit—The lack of standardized, sensitive, and minimally invasive biomarkers for early mitochondrial dysfunction limits timely risk identification. While creatine levels in biofluids (serum, plasma, urine, saliva) and tissues (skeletal muscle, brain) can be measured, they are rarely integrated into routine clinical diagnostics.
2. Predictive deficit—Creatine-based biomarkers are largely absent from current multi-omics models and computational prediction frameworks, reducing precision in risk stratification.
3. Personalization deficit—Despite variability in creatine metabolism influenced by genetic polymorphisms (e.g., *SLC6A8*, *CKM*, *GATM*), age, sex, diet, and comorbidities, current supplementation regimens remain “one-size-fits-all,” leading to suboptimal therapeutic outcomes.
4. Translational deficit—Advanced delivery systems such as intranasal administration, nanoparticle encapsulation, and slow-release matrices remain under-validated for clinical use, limiting targeted bioavailability enhancements.

Addressing these gaps positions creatine as both a modifiable therapeutic factor capable of restoring mitochondrial function and a quantifiable diagnostic marker of mitochondrial health and treatment responsiveness. Functional testing approaches—such as creatine loading protocols or dynamic magnetic resonance spectroscopy (MRS)-based assessments—may provide real-time evaluation of mitochondrial reserve capacity and inform individualized dosing strategies. From a health systems perspective, creatine offers scalability, affordability, and an established safety profile, making it a promising adjunct in public health strategies aimed at preventing chronic disease, mitigating functional decline, and extending healthspan. The integration of creatine biomarkers with digital health tools, wearable biosensors, and adaptive dosing algorithms could further enhance personalization and patient engagement in preventive care. In this mini review, we synthesize current evidence on creatine’s biochemical and cellular effects on mitochondrial function, evaluate its diagnostic potential within PPPM-aligned multi-level frameworks, and outline strategies for personalized supplementation. By addressing current diagnostic, predictive, and therapeutic deficits, we present a systems-level approach to repositioning creatine from a sports nutrition supplement to a clinically relevant tool in mitochondrial medicine.

## Biochemical and cellular roles of creatine in mitochondrial function

The classical physiological role of creatine centers on its participation in the reversible conversion to phosphocreatine, catalyzed by both mitochondrial and cytosolic isoforms of creatine kinase (CK), which together function to buffer intracellular ATP levels [10]. The mitochondrial creatine kinase isoform (mtCK), in particular, forms a multienzyme complex with the adenine nucleotide translocator (ANT) and the voltage-dependent anion channel (VDAC), effectively coupling mitochondrial ATP synthesis to cytosolic ATP utilization [11]. This phosphagen system facilitates the rapid regeneration and spatial distribution of ATP, a process critically important in tissues with fluctuating and elevated energy demands, such as contracting skeletal muscle and activated neurons. The integrity of this system is essential for maintaining cellular energy homeostasis, and disruptions in creatine availability and/or CK activity are increasingly recognized as pathogenic features in both metabolic and neurological disorders [12]. Deficiencies in CK isoforms or impaired creatine transport and synthesis result in inadequate ATP buffering capacity, leading to energetic deficits and subsequent cellular dysfunction. Animal and clinical studies have shown that genetic ablation, disease-related impairment, or dietary depletion of creatine leads to phenotypic manifestations characterized by diminished muscle contractility, cognitive dysfunction, and increased susceptibility to metabolic stress [13–16]. These findings highlight the centrality of the creatine-phosphocreatine system in sustaining mitochondrial bioenergetics and overall cellular viability, particularly under conditions of high or fluctuating energy demand.

MtCK functions not only as a critical metabolic relay but also as a structural stabilizer of mitochondrial membranes through its specific interaction with cardiolipin, a key phospholipid of the inner mitochondrial membrane [17]. This interaction supports the preservation of mitochondrial ultrastructure, including cristae integrity, and plays a protective role in preventing the release of cytochrome c under conditions of oxidative or metabolic stress [18]. MtCK is a central component of a supramolecular complex that physically couples mitochondrial ATP production to cytosolic phosphorylation reactions via the creatine/phosphocreatine shuttle. This coupling facilitates efficient energy transfer and buffering, particularly during periods of high and fluctuating energy demands. Importantly, mtCK contributes to the maintenance of the ADP/ATP ratio, which is essential for sustaining oxidative phosphorylation and preventing mitochondrial dysfunction. A decline in mtCK activity or expression—whether due to genetic, pathological, or nutritional factors—has been associated with impaired mitochondrial respiration, increased ROS production, and heightened susceptibility to cellular stressors [19]. The availability of creatine is a key determinant of mtCK function. Without sufficient intracellular creatine, mtCK cannot effectively catalyze the formation of phosphocreatine or buffer ATP levels, ultimately compromising mitochondrial energy dynamics. This becomes especially relevant in disease states or aging, where endogenous creatine synthesis or uptake may be impaired [20]. MtCK dysfunction has been implicated in several pathological conditions characterized by mitochondrial fragmentation, membrane instability, and apoptosis [21]. Conversely, experimental studies have shown that overexpression of mtCK enhances cellular resilience to oxidative stress, maintains mitochondrial integrity, and improves functional recovery following ischemic injury [22]. These findings highlight the therapeutic potential of preserving mtCK function and ensuring adequate creatine availability as part of strategies aimed at stabilizing mitochondrial performance and preventing bioenergetic failure.

Emerging evidence suggests that creatine availability also plays a regulatory role in mitochondrial biogenesis, primarily through the activation of AMP-activated protein kinase (AMPK) and its downstream effector peroxisome proliferator-activated receptor gamma coactivator-1 alpha (PGC-1α), a key transcriptional regulator of mitochondrial gene expression [23, 24]. Creatine loading has been shown to increase mitochondrial DNA (mtDNA) copy number, enhance expression of oxidative phosphorylation (OXPHOS) genes, and elevate respiratory capacity in both skeletal muscle and neural tissues—two energetically demanding and mitochondria-rich systems [25, 26]. In addition to promoting mitochondrial biogenesis, creatine appears to influence mitochondrial dynamics by modulating the balance between fission and fusion processes [27], which are essential for mitochondrial quality control, adaptation to energetic stress, and inter-organelle communication. These effects contribute to the maintenance of a functional and interconnected mitochondrial network, particularly in the context of metabolic challenges or age-associated decline. Creatine may further interface with other cellular longevity pathways, including sirtuin signaling and NAD⁺ metabolism, potentially amplifying its effects on mitochondrial health and cellular resilience [28]. Of particular interest is creatine’s proposed role as an epigenetic modulator: by affecting the availability of *S*-adenosylmethionine (SAM)—a major methyl donor consumed during creatine biosynthesis—creatine supplementation may indirectly influence global and gene-specific methylation patterns [29, 30]. This epigenetic regulation could impact the transcription of genes involved in mitochondrial maintenance, oxidative stress response, and cellular differentiation. Taken together, these findings support the concept that creatine not only serves as a bioenergetic substrate but also acts as a signaling molecule that orchestrates mitochondrial biogenesis, dynamics, and epigenetic adaptation—positioning it as a multifaceted agent for enhancing mitochondrial health within the framework of predictive, preventive, and personalized medicine.

## Creatine and oxidative stress in mitochondria

Creatine exerts both direct and indirect antioxidant effects (for a detailed review see Ref. [31]. While its direct free radical scavenging activity is modest compared to classical antioxidants, creatine nonetheless contributes to redox homeostasis by reducing reactive oxygen species (ROS) and preserving mitochondrial integrity. One of its key indirect mechanisms involves supporting the glutathione antioxidant system. By sustaining cellular ATP levels through the phosphocreatine system, creatine reduces the metabolic burden on NADPH-dependent pathways, thereby sparing NADPH for the regeneration of reduced glutathione [32]. This mechanism attenuates mitochondrial oxidative damage, protein nitration, and lipid peroxidation. Evidence from preclinical and clinical studies consistently demonstrates that creatine supplementation lowers biomarkers of oxidative stress across various models and populations [33–37]. These antioxidant effects are particularly significant in pathologies such as neurodegenerative diseases and ischemia–reperfusion injury, where oxidative imbalance plays a central pathogenic role [38, 39]. In addition to mitigating oxidative stress, creatine modulates inflammatory signaling pathways. Supplementation has been shown to downregulate the expression of pro-inflammatory cytokines such as tumor necrosis factor-alpha (TNF-α) and interleukin-6 (IL-6) in models of systemic inflammation and neuroinflammation [40, 41].

In addition, creatine has been increasingly recognized for its role in regulating mitochondrial permeability transition pore (mPTP) dynamics, a pivotal event in the initiation of apoptosis and necrosis [42]. Specifically, creatine contributes to the stabilization of mitochondrial membrane potential and sustains ATP buffering capacity under conditions of metabolic and oxidative stress [43]. These actions collectively attenuate calcium-induced mitochondrial swelling and membrane depolarization, thereby enhancing cellular resilience and survival. Evidence from in vitro and in vivo models indicates that creatine supplementation can delay the opening of the mPTP and significantly reduce cell death in response to oxidative insults [23, 44]. Such findings underscore creatine’s therapeutic potential in preserving mitochondrial integrity in acute pathologies characterized by bioenergetic failure, including ischemic stroke, myocardial infarction, and traumatic brain injury [45, 46]. Moreover, recent proteomic and mechanistic studies suggest that creatine availability may modulate the expression and phosphorylation states of key regulatory proteins of the mPTP, such as cyclophilin D and the voltage-dependent anion channel (VDAC) [47, 48]. These findings position creatine not solely as a metabolic buffer, but as an active signaling modulator of mitochondrial cell death pathways.

## Creatine as a biomarker within multi-level diagnostic frameworks in PPPM

Emerging evidence supports the use of creatine concentrations in various biological matrices—including serum, plasma, saliva, and tissue-specific compartments such as skeletal muscle and the brain—as a non-invasive or minimally invasive component within multi-level diagnostic frameworks aimed at evaluating muscle mass, metabolic status, and neurological function. Notably, tissue creatine levels can be quantified in vivo using advanced imaging modalities such as magnetic resonance spectroscopy (MRS), offering valuable insights into organ-specific bioenergetics (for a detailed review, see Ref. [49]. Circulating and tissue creatine concentrations have been shown to correlate with multiple physiological and pathological states. These include age-related decline in creatine levels [50], neuromuscular disorders such as muscular dystrophies and mitochondrial myopathies [51], cardiometabolic conditions including heart failure and insulin resistance [52], and neurological pathologies such as brain tumors [53] and post-viral fatigue syndromes [16]. These associations highlight the diagnostic potential of creatine in a broad spectrum of chronic and degenerative diseases, particularly those with a mitochondrial component.

Among emerging modalities, salivary creatine analysis offers a particularly promising platform for rapid, cost-effective, and non-invasive monitoring in both clinical and athletic settings [54]. Efforts are underway to establish standardized reference ranges for creatine in saliva, blood, and tissue compartments, incorporating adjustments for age, sex, and physiological status to enhance clinical interpretability [55]. The integration of advanced analytical technologies—such as capillary electrophoresis-mass spectrometry (CE-MS) and high-resolution MRS—is further improving the sensitivity, specificity, and spatial resolution of creatine quantification. These tools are enabling the identification of subtle metabolic shifts and region-specific creatine depletion patterns that may precede clinical symptoms, thereby supporting the early stages of disease prediction and stratification.

## Clinical illustration 1: creatine profiling in long COVID

In alignment with the predictive dimension of PPPM, creatine profiling can serve as a clinically relevant tool for patient phenotyping, early risk detection, and individualized outcome prediction. A recent case series [16] highlights how this approach may address a critical unmet need—the lack of objective, non-invasive biomarkers to guide diagnosis, prognosis, and management in post-viral fatigue syndromes such as long COVID. The series involved a cohort of patients (*n* = 19) who had experienced persistent fatigue, exertional intolerance, and musculoskeletal pain for more than three months following confirmed SARS-CoV-2 infection. Standard laboratory tests, cardiopulmonary evaluations, and structural brain MRI yielded largely unremarkable findings, leaving the underlying pathophysiology and prognosis uncertain. To assess tissue bioenergetics, all patients underwent creatine quantification using 1.5 T single-voxel proton MRS in both the vastus medialis muscle and selected brain regions (thalamus, cortical gray matter, and white matter). Compared with established reference ranges from healthy populations, the patients displayed markedly reduced total creatine concentrations in skeletal muscle and across multiple brain structures. Notably, muscle creatine levels showed a consistent inverse correlation with the severity of myalgia, suggesting that creatine depletion may contribute to impaired ATP buffering, mitochondrial dysfunction, and symptom burden. From a PPPM perspective, these findings illustrate the potential of creatine profiling to fill several gaps in current care: (1) *Unmet diagnostic need*: offers a quantifiable, non-invasive biomarker of tissue bioenergetics where conventional clinical tests fail to reveal abnormalities; (2) *Predictive value*: Links baseline creatine depletion with greater symptom severity, supporting its role as a prognostic indicator for disease course; and (3) *Personalization potential*: Enables stratification of patients according to underlying bioenergetic deficits, facilitating tailored therapeutic strategies—such as creatine supplementation or targeted rehabilitation—rather than applying uniform post-viral fatigue protocols. Although the study was cross-sectional and limited by modest sample size and potential confounding factors (e.g., unmeasured physical activity levels, inflammatory status), it demonstrates how MRS-based creatine profiling could be integrated into multi-parametric diagnostic frameworks. In combination with other metabolic, inflammatory, and functional biomarkers, longitudinal monitoring of creatine status may improve the precision of disease trajectory predictions, optimize treatment selection, and ultimately better meet the needs of patients with complex, multisystem disorders like long COVID.

In mitochondrial encephalomyopathies and other disorders of oxidative phosphorylation, alterations in creatine levels—measured in tissue, urine, or blood—have been shown to correlate with disease severity and therapeutic responsiveness [56]. These creatine profiles serve as valuable biochemical indicators of cellular bioenergetic status, particularly in clinical contexts marked by impaired mitochondrial ATP synthesis and disrupted phosphocreatine buffering systems. MRS enables the non-invasive quantification of creatine concentrations in anatomically distinct tissues such as the brain and myocardium, offering spatially resolved insights into mitochondrial function and regional energetic deficits [57]. Within this framework, decreased creatine-to-choline and creatine-to-N-acetylaspartate (NAA) ratios are being actively investigated as surrogate markers of neuronal and muscular integrity, as well as functional proxies for mitochondrial capacity [58]. These MRS-derived metrics may enhance conventional diagnostic workflows by complementing clinical examination and biochemical testing, particularly in heterogeneous or progressive mitochondrial pathologies where early detection is challenging. Dynamic changes in creatine concentrations—whether in response to nutritional interventions, cofactor therapy, or gene-targeted treatments—may serve as early biomarkers of therapeutic efficacy. In many cases, alterations in tissue or systemic creatine levels precede measurable improvements in clinical symptoms or functional capacity, underscoring their potential role in monitoring disease progression and treatment responsiveness over time. Moreover, integrating creatine-based biomarkers with multi-omics data—including genomic variants (e.g., SLC6A8, GAMT), transcriptomic profiles, and complementary metabolic markers such as lactate, pyruvate, and acylcarnitine—may substantially improve diagnostic precision and enable the subtyping of complex mitochondrial and metabolic disorders [59]. Such integrative approaches align with the principles of PPPM, facilitating the development of tailored therapeutic strategies based on a patient's unique bioenergetic phenotype. Inter-individual variability in creatine kinetics—modulated by factors such as age, sex, dietary intake, hormonal status, and genetic background—further underscores the relevance of creatine as a component of personalized metabolic profiling [9, 55]. Creatine loading tests, in particular, may offer a functional means of assessing mitochondrial reserve capacity and predicting responsiveness to supplementation strategies. Ongoing work in computational modeling and metabolomics is focused on developing algorithms that integrate creatine dynamics with other physiological parameters to support individualized risk stratification in neuromuscular and metabolic disease. Ultimately, personalized creatine assessment—enabled by emerging technologies such as portable MRS, wearable biosensors, and digital health platforms—holds promise for guiding lifestyle interventions, optimizing nutritional support, and informing early therapeutic decisions.

## Creatine supplementation in disease prevention and therapy

Neurodegenerative disorders such as Alzheimer’s disease (AD), Parkinson’s disease (PD), and Huntington’s disease (HD) are typified by progressive neuronal loss, disrupted mitochondrial dynamics, and sustained oxidative stress, all of which contribute to the deterioration of cognitive and motor function [60]. Mitochondrial dysfunction, including impaired ATP production, reduced membrane potential, and increased susceptibility to apoptosis, is a central feature of these diseases and represents a promising target for therapeutic intervention. Preclinical studies have demonstrated that creatine supplementation can mitigate mitochondrial impairment across several neurodegenerative models. In rodent models of HD, creatine has been shown to improve cognitive performance and prolong survival, likely by enhancing ATP buffering and reducing oxidative damage [61]. Similarly, in PD models, creatine preserves dopaminergic neurons and improves mitochondrial resilience in the substantia nigra [62]. These effects are attributed to creatine’s multifaceted actions on mitochondrial energy metabolism, membrane stabilization, and apoptosis regulation [63]. Clinical trial outcomes, however, have been variable. Some studies report beneficial effects of creatine on working memory, muscular strength, and fatigue in early-stage PD and HD patients, while others fail to demonstrate significant improvements in more advanced stages of disease (for comprehensive reviews, see Refs. [64] and [65]). This inconsistency likely reflects the inherent heterogeneity in disease progression, baseline mitochondrial reserve, and individual differences in creatine uptake and metabolism. From the perspective of PPPM, these findings underscore the importance of patient stratification in designing future intervention trials. Creatine is unlikely to be uniformly effective across all stages or subtypes of neurodegeneration. Instead, its therapeutic potential may be optimized in carefully selected subpopulations—particularly those in early disease stages with measurable deficits in mitochondrial function or energy buffering capacity. Incorporating biomarkers of mitochondrial health, genetic profiles, and neuroimaging data could further enhance the precision of such interventions. Future clinical trials should adopt stratified designs aligned with PPPM principles, integrating creatine as part of a broader, mitochondria-targeted therapeutic strategy aimed at neuroprotection and disease modification in early-phase neurodegenerative disorders.

In type 2 diabetes mellitus (T2DM) and metabolic syndrome, mitochondrial dysfunction is a key pathogenic driver contributing to impaired insulin signaling, ectopic lipid accumulation, and chronic low-grade inflammation [66]. These alterations in cellular bioenergetics reduce metabolic flexibility and exacerbate insulin resistance across multiple tissues, including skeletal muscle, liver, and adipose tissue. Preclinical studies have shown that creatine supplementation can attenuate metabolic impairments in high-fat diet-induced models of insulin resistance. Specifically, creatine has been demonstrated to improve glucose tolerance, enhance insulin signaling via the Akt and AMPK pathways, and reduce hepatic steatosis by modulating lipid metabolism and mitochondrial function [67]. In human studies, creatine supplementation—particularly when combined with structured exercise training—has been associated with improvements in fasting plasma glucose, glycated hemoglobin (HbA1c), and central adiposity, as reflected in reductions in waist circumference [68]. These effects underscore creatine’s potential as a mitochondrial modulator in the management of metabolic dysregulation. In the context of cardiovascular disease (CVD), where myocardial energy deficiency and impaired calcium handling are central to disease progression, creatine plays a vital role in supporting cardiac energetics. By facilitating ATP turnover in cardiomyocytes and stabilizing intracellular calcium dynamics, creatine can help maintain contractile function and myocardial resilience (for a detailed review, see Ref. [69]). In animal models of heart failure, creatine supplementation has been shown to improve cardiac output, attenuate myocardial fibrosis, and increase survival rates [70]. Clinical evidence, while still emerging, suggests that creatine may enhance functional capacity and quality of life in patients with heart failure with reduced ejection fraction [71]. These benefits are particularly relevant in advanced heart failure phenotypes where mitochondrial impairment and energy depletion contribute to symptom burden and disease progression. Nevertheless, larger, adequately powered randomized controlled trials are needed to confirm these findings and define optimal dosing strategies, timing of intervention, and patient subgroups most likely to benefit. Collectively, these findings support the integration of creatine into personalized approaches for the prevention and management of metabolic and cardiovascular disorders, particularly when guided by biomarkers of mitochondrial function and energy metabolism within the framework of PPPM.

Mitochondrial decline is a well-recognized hallmark of aging and contributes significantly to sarcopenia—the progressive loss of skeletal muscle mass, strength, and function observed in older adults [72]. Sarcopenia is associated with an increased risk of falls, frailty, disability, and reduced quality of life. In this context, creatine supplementation has been extensively studied as a nutritional strategy to counteract age-related musculoskeletal decline. Clinical trials have consistently shown that creatine supplementation enhances muscle strength, increases lean body mass, and improves performance on standardized functional assessments, including gait speed, chair rise time, and balance tests. Notably, these benefits are more pronounced when creatine is combined with resistance training, as demonstrated in multiple randomized controlled trials and meta-analyses [73]. Furthermore, some studies have reported that creatine use may contribute to fall prevention and improved mobility in elderly individuals at risk for functional decline [74]. Emerging observational and interventional data suggest that creatine may also play a role in preserving broader aspects of physical function and quality of life in aging populations [75]. Although long-term studies are still needed, the available evidence supports the safety, feasibility, and clinical relevance of creatine supplementation as part of a comprehensive strategy to maintain musculoskeletal health in older adults. Within the framework of PPPM, creatine represents a promising, low-cost intervention to help delay the onset of frailty and functional impairment, particularly when tailored to individual risk profiles and integrated into lifestyle-based preventive care models.

## Creatine and personalized supplementation strategies

Genetic variability within the creatine biosynthesis and transport pathways significantly influences individual responses to creatine supplementation. Polymorphisms in genes encoding enzymes and transporters essential for creatine metabolism—such as *L-*arginine:glycine amidinotransferase (*AGAT*), guanidinoacetate methyltransferase (*GAMT*), and the creatine transporter *SLC6A8*—have been identified as key determinants of inter-individual differences in creatine homeostasis and therapeutic efficacy [76]. A well-characterized example is X-linked creatine transporter deficiency, caused by mutations in *SLC6A8*, which leads to cerebral creatine deficiency, intellectual disability, seizures, and developmental delays [77]. In these cases, conventional oral creatine monotherapy is largely ineffective due to impaired transport across the blood–brain barrier, prompting the exploration of alternative delivery methods such as intranasal supplementation and gene-based therapies [78, 79]. Beyond rare genetic disorders, substantial inter-individual variability in response to creatine supplementation is observed in the general population. This variability is shaped by a complex interplay of biological and lifestyle factors, including baseline tissue creatine concentrations, muscle fiber type composition, bone remodeling capacity, neuro-energetic status, habitual dietary creatine intake, sex, age, and physical activity level [80]. Such heterogeneity underscores the necessity of personalized supplementation protocols tailored to individual physiological profiles and health objectives. Within the framework of PPPM, genotyping may serve as a valuable tool for stratifying individuals based on their capacity to synthesize, transport, and utilize creatine. When integrated with phenotypic assessments—such as brain creatine quantification via MRS—this approach can enhance the precision and efficacy of creatine-based interventions, particularly in clinical and aging populations.

While creatine monohydrate (CrM) remains the gold standard for oral supplementation due to its robust safety and efficacy profile, alternative formulations have been developed to improve solubility, absorption, and tissue-specific delivery. These include creatine hydrochloride, creatine ethyl ester, buffered creatine, and a variety of synthetic analogs, each exhibiting differing pharmacokinetic and pharmacodynamic properties (for a detailed review, see Ref. [81]). In parallel, novel delivery technologies such as liposomal encapsulation, solid lipid nanoparticles, and PEGylated creatine are being considered for their potential to enhance systemic bioavailability and bypass gastrointestinal degradation. Of particular interest for neurological applications are nasal and transmucosal delivery routes, which may bypass first-pass metabolism and the blood–brain barrier, thereby enabling more efficient creatine delivery to central nervous system tissues [82]. These innovations are especially promising for treating conditions characterized by impaired creatine transport, including creatine deficiency syndromes and neurodegenerative disorders. In parallel, advances in digital health are paving the way for individualized monitoring and management of creatine supplementation. Wearable devices and mobile health applications that integrate physiological metrics (e.g., fatigue indices, sleep quality) with dietary tracking and dosing algorithms offer opportunities for real-time feedback and dynamic personalization. The development of biosensors capable of measuring creatine concentrations in saliva, sweat, or urine would further support at-home monitoring and adaptive dosing strategies, particularly in outpatient or preventive care settings. Machine learning and artificial intelligence (AI) platforms are poised to play a central role in analyzing these complex, multidimensional datasets—linking genotypic, phenotypic, and environmental information to refine dose–response models and optimize outcomes. Together, these innovations represent a convergence of nutrigenomics, precision supplementation, and digital health technologies, positioning creatine as a cornerstone of personalized bioenergetic support in both preventive and therapeutic domains of modern medicine.

## Clinical illustration 2: personalized creatine supplementation in post-viral fatigue syndrome

A recent parallel-group, randomized, double-blind, placebo-controlled trial [83] evaluated the effects of six months of creatine monohydrate supplementation (4 g/day) in twelve adults aged 18–65 years with persistent post-COVID-19 fatigue syndrome (PVFS)—a complex condition characterized by mitochondrial dysfunction despite unremarkable results on standard clinical assessments. The intervention produced notable benefits across three domains: tissue bioenergetics, symptom resolution, and functional capacity. Compared with baseline, the creatine group demonstrated significant increases in tissue creatine concentrations—most prominently in the vastus medialis muscle and right parietal white matter—at both 3- and 6-month follow-ups, with changes significantly greater than those observed in the placebo group. Clinically, general fatigue scores improved after just three months of supplementation, and by six months, participants reported substantial relief from multiple PVFS–associated symptoms, including ageusia, breathing difficulties, body aches, headache, and problems with concentration. Exercise tolerance, measured as time-to-exhaustion during walking tests, also improved. Cohen’s effect-size analyses further supported improvements in mental fatigue and increased creatine levels across several brain regions. From a PPPM perspective, this trial addresses key gaps: (1) *Diagnostic precision and predictive value*—demonstrating that measurable tissue creatine replenishment correlates with symptom improvement, supporting creatine as a diagnostic and prognostic biomarker of bioenergetic dysfunction in PVFS; (2) *Personalization of care*—allowing targeted, data-driven interventions for patients with documented creatine deficits, rather than relying on nonspecific fatigue management; and (3) *Therapeutic efficacy and safety*—showing tangible, clinically relevant benefits with minimal adverse effects (only one case of mild, transient nausea). This study exemplifies the theranostic potential of creatine within PPPM, bridging diagnostics and therapy by using creatine levels both as a marker of mitochondrial health and as a guide for individualized supplementation. Integrating such protocols could shift post-viral fatigue management from empirical approaches to mechanistically grounded, personalized medicine.

## Future perspectives

Despite robust preclinical and clinical data supporting creatine’s role as a mitochondrial-targeted agent, several critical gaps must be addressed to enable its full integration into the framework of PPPM. First, large-scale, longitudinal studies are needed to evaluate the long-term safety and efficacy of creatine supplementation across diverse populations, including older adults, pediatric cohorts, and individuals with chronic or metabolic diseases. Second, the establishment of standardized reference ranges for creatine concentrations in biofluids—such as plasma, urine, and saliva—is essential. These ranges should be adjusted for age, sex, and ethnicity to improve diagnostic precision within multi-level diagnostic frameworks aimed at assessing mitochondrial viability. Third, digital health integration remains underdeveloped; the validation of biosensors and wearable technologies capable of real-time creatine monitoring is necessary to support personalized dosing and adaptive supplementation strategies. Fourth, there is growing interest in exploring creatine’s synergistic potential with other mitochondria-targeting compounds such as NAD⁺ precursors, coenzyme Q10, and pyrroloquinoline quinone (PQQ), which may enhance cellular resilience and energy metabolism. Finally, emerging evidence suggests that creatine may influence oncologic processes, including tumor metabolism, immune modulation, and cancer cachexia [84]. Investigating its role in mitochondrial reprogramming and immunometabolic regulation could uncover novel applications in oncology and expand its therapeutic reach within PPPM.

## Conclusions and expert recommendations in the framework of 3PM

**Fig. 1:**
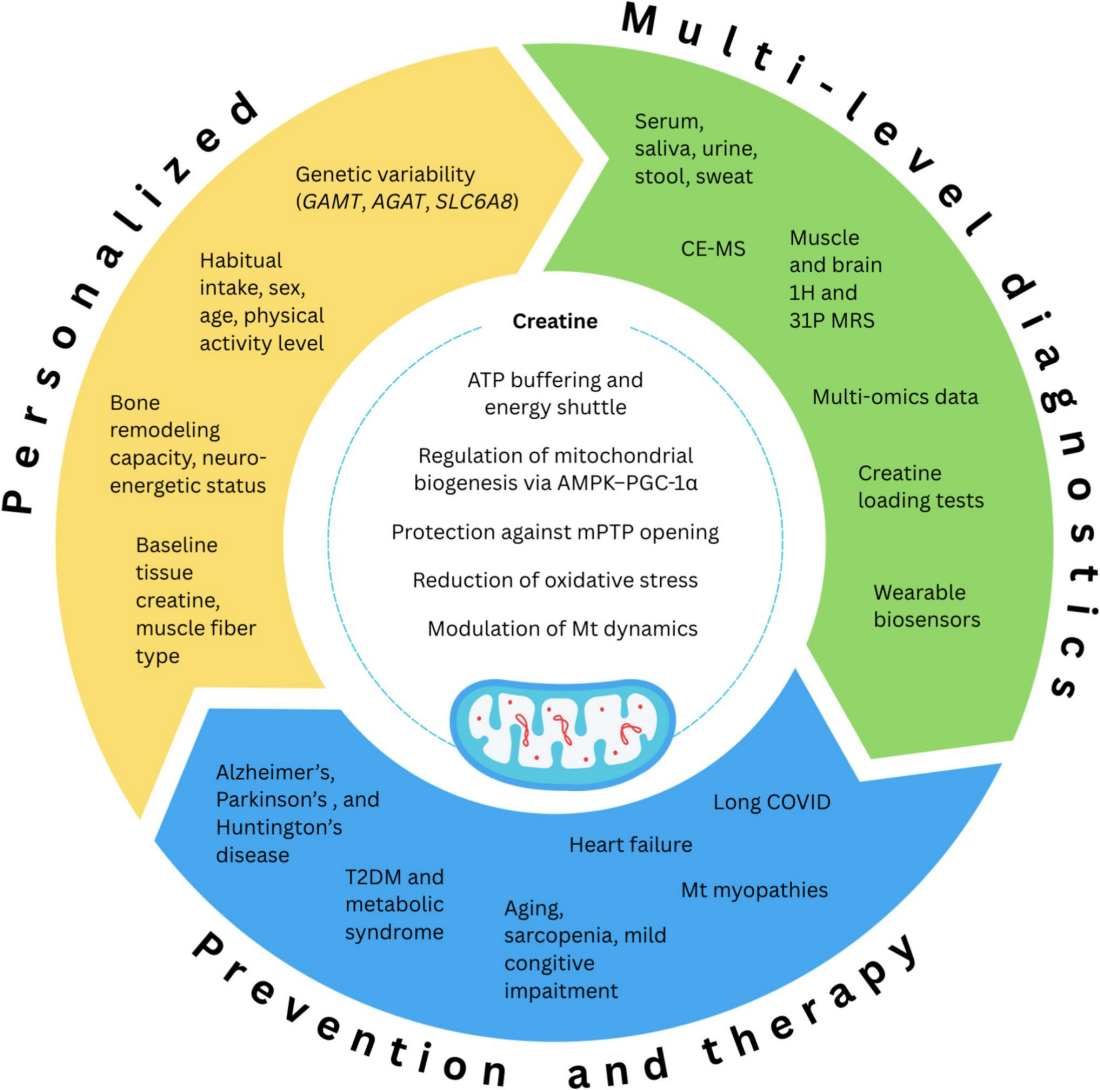
Continuum of creatine as a mitochondrial theranostic in 3PM context. *Abbreviations*: AGAT, *L-*arginine:glycine amidinotransferase; AMPK, adenosine 5′-monophosphate-activated protein kinase; ATP, adenosine triphosphate; CE-MS, capillary electrophoresis-mass spectrometry; GAMT, guanidinoacetate methyltransferase; mPTP, mitochondrial permeability transition pore; MRS, magnetic resonance spectroscopy; Mt, mitochondrial; PGC-1α, peroxisome proliferator-activated receptor gamma coactivator 1-alpha; SLC6A8, solute carrier family 6 member 8; T2DM, type 2 diabetes mellitus

Creatine is increasingly recognized as a critical bioenergetic compound and a mitochondria-targeted theranostic agent that aligns closely with the core principles of PPPM (Fig. 1). Its pleiotropic biological actions—including the stabilization of cellular energy homeostasis, regulation of redox balance, and modulation of mitochondrial dynamics and apoptotic pathways—highlight its broad therapeutic potential. Given that mitochondrial dysfunction represents a common underlying mechanism across a wide spectrum of chronic, degenerative, and age-related diseases, creatine-based strategies may serve as a cornerstone for personalized health maintenance and early disease interception within PPPM frameworks.

From a predictive standpoint, profiling creatine concentrations in biofluids and tissues offers a minimally invasive means to detect early bioenergetic disturbances, evaluate mitochondrial reserve capacity, and forecast individual susceptibility to metabolic dysfunction. These insights support the development of personalized risk profiles and inform the timing, intensity, and modality of preventive interventions. Furthermore, creatine levels can be integrated into digital health platforms and biomarker dashboards, enabling continuous physiological monitoring and the deployment of real-time early warning systems.

Preventive applications of creatine are supported by its well-established safety profile, affordability, and broad adaptability across age groups and clinical populations—from congenital creatine deficiency syndromes to sarcopenia and neurodegeneration. Personalized supplementation strategies, tailored to individual phenotypic characteristics, genetic polymorphisms (e.g., *SLC6A8*, *CKM*, *GATM*), comorbid conditions, and lifestyle factors, are essential to optimize therapeutic outcomes while minimizing adverse effects. Functional assessments such as creatine loading tests and advanced tissue imaging (e.g., magnetic resonance spectroscopy) may offer dynamic, patient-specific evaluations of mitochondrial responsiveness and guide individualized preventive protocols.

In the context of personalized medicine, creatine confers a unique dual role: it serves both as a modifiable regulator of mitochondrial health and as a measurable biomarker of therapeutic efficacy. This dual functionality facilitates feedback-driven treatment adjustment, consistent with the adaptive and iterative nature of personalized healthcare. Moreover, creatine may synergize with other mitochondrial-targeted therapies—including coenzyme Q10, nicotinamide riboside, and exercise mimetics—opening new avenues for multimodal and combinatorial interventions tailored to the patient’s specific bioenergetic phenotype.

To fully operationalize creatine within PPPM, several expert-driven priorities must be addressed. First, there is an urgent need to develop and standardize diagnostic protocols, including the establishment of validated reference ranges for creatine across multiple biological matrices, with appropriate stratification by age, sex, ethnicity, and physiological state. Second, creatine biomarkers should be incorporated into multi-omics platforms—combining genomics, metabolomics, imaging, and exposomics—to facilitate deep phenotyping and improve risk stratification in complex disorders. Third, well-designed longitudinal and randomized controlled trials are needed to define optimal dosing regimens, identify responder subpopulations, and determine long-term clinical outcomes. Finally, the adoption of digital health innovations—including wearable biosensors and AI-assisted decision-support tools—may enhance the scalability, personalization, and cost-effectiveness of creatine-centered care models.

Looking ahead, the convergence of nutritional science, high-resolution biomarker technologies, and digital infrastructure offers a transformative opportunity to reposition creatine as a foundational agent in metabolic and mitochondrial medicine. Its safety, low cost, and extensive mechanistic validation render creatine particularly suitable for integration into precision health strategies aimed at enhancing cellular resilience, delaying disease onset, and promoting healthy aging. In this evolving context, creatine is poised to transcend its traditional role in sports nutrition and emerge as a clinically relevant, systems-based solution in the delivery of proactive, personalized medicine.

## Data Availability

No datasets were generated or analysed during the current study.
